# 4108 Artificial Intelligence-Based Quantification of the General Movement Assessment Using Center of Pressure Patterns in Healthy Infants

**DOI:** 10.1017/cts.2020.324

**Published:** 2020-07-29

**Authors:** Tara Lynn Johnson, Namarta Kapil, Alexa Escapita, Bittu Majmudar, Safeer Siddicky, Junsig Wang, Erin Mannen

**Affiliations:** 1 University of Arkansas for Medical Sciences

## Abstract

OBJECTIVES/GOALS: One in six children in the U.S. has a Neurodevelopmental Disability (NDD). Prechtl’s General Movement Assessment (GMA) is a qualitative predictor of early motor dysfunction. However, no quantitative biomechanical assessment exists to more accurately identify all patients with NDD. METHODS/STUDY POPULATION: With UAMS IRB approval, as part of a larger study, healthy infants were filmed while lying supine on a force plate for 2 minutes. We studied 12 healthy full-term infants (gestational age: 38.9±1.5 weeks, age: 2.1-7.0 months; 7M, 5F; length: 64.0±5.2 cm; weight: 7.2±1.3 kg). Within our data set there were 3 infants transitioning to fidgety period (≤3 months), 4 in the fidgety period, (3-5 months), and 5 that matured beyond fidgety period (>5 months). Center of pressure (COP) path-lengths were gathered from the force plate at 1000 Hz. We grouped our data with K-means clustering and performed statistical analysis with ANOVA. RESULTS/ANTICIPATED RESULTS: We divided our data into 3 distinct clusters. The first group contained infants with moderate variability of movements which included 2 infants between 3 and 5 months and 2 infants slightly outside of this range. The second group, with mild variability in movements, included 4 infants between 2 and 3 months as well as 2 infants just older than 5 months. The third group, with little variability in movements, included 2 infants older than 5 months. A GMA reader (TJ) qualitatively confirmed these findings with video footage. Using a threshold of p<0.05, data sets within the clusters were similar and significantly different from other clusters. DISCUSSION/SIGNIFICANCE OF IMPACT: Fidgety infants have greater variability in COP patterns than their mature counterparts. We anticipate additional COP measurements will correspond with qualitative GMA analyses. Artificial Intelligence-based quantification of the GMA may be useful in earlier detection or prediction of NDD outcomes.

